# Stochastic Microsensors Based on Carbon Nanotubes for Molecular Recognition of the Isocitrate Dehydrogenases 1 and 2

**DOI:** 10.3390/nano12030460

**Published:** 2022-01-28

**Authors:** Raluca-Ioana Stefan-van Staden, Catalina Cioates Negut, Sorin Sebastian Gheorghe, Paula Sfirloaga

**Affiliations:** 1 Laboratory of Electrochemistry and PATLAB, National Institute of Research for Electrochemistry and Condensed Matter, 202 Splaiul Independentei St., 060021 Bucharest, Romania; 2 Faculty of Applied Chemistry and Material Science, Politehnica University of Bucharest, 060021 Bucharest, Romania; sebastiangheorghe09@yahoo.com; 3 National Institute of Research for Electrochemistry and Condensed Matter, Dr. Aurel Paunescu Podeanu 144, 300569 Timisoara, Romania; paulasfirloaga@gmail.com

**Keywords:** stochastic microsensors, isocitrate dehydrogenase 1, isocitrate dehydrogenase 2, carbon nanotubes

## Abstract

Two three-dimensional (3D) stochastic microsensors based on immobilization of protoporphyrin IX (PIX) in single-walled carbon nanotubes (SWCNT) and multi-walled carbon nanotubes (MWCNT) decorated with copper (Cu) and gold (Au) nanoparticles were designed and used for the molecular recognition of isocitrate dehydrogenase 1 (IDH1) and isocitrate dehydrogenase 2 (IDH2) in biological samples (brain tumor tissues, whole blood). The linear concentration ranges obtained for the molecular recognition and quantification of IDH1 and IDH2 were: IDH1 (1 × 10^−5^–1 × 10^2^ ng mL^−1^) and IDH2 (5 × 10^−8^ − 5 × 10^2^ ng mL^−1^). The limits of quantification obtained using the proposed microsensors were: 10 fg mL^–1^ for IDH1 and 5 × 10^−3^ fg mL^−1^ for IDH2. The highest sensitivities were obtained for the microsensor based on MWCNT. High recoveries versus enzyme-linked immunosorbent assay (ELISA) standard method were recorded for the assays of IDH1 and IDH2, all values being higher than 99.00%, with relative standard deviations (RSD) lower than 0.10%.

## 1. Introduction

Enzyme and gene assays play a very important role in cancer diagnosis. There are two genes—human isocitrate dehydrogenase (IDH) isoforms—which are homodimer isoenzymes: IDH1 found in cytoplasm and peroxisomes, and IDH2 in mitochondria. IDH1 and IDH2 play a very important role in the diagnosis of brain cancer [1,2,3,4,5,6]. Accordingly, they can be used as biomarkers for the rapid diagnosis of brain cancer/gliomas, which are encountered frequently in highly developed countries and have the worst prognosis among solid cancers. Diffuse gliomas are the most common primary brain tumors found in adults, affecting approximately 20,000 people annually in the United States [7].

To date, polymerase chain reaction (PCR) and deoxyribonucleic acid (DNA) sequencing are the main techniques used for the assay of IDH1 and IDH2, e.g., DNA pyrosequencing was proposed for the assay of IDH1 and IDH2 [8]. A multiplex-based bead assay [9] and a fluorescence method [10] were also proposed for the assay of IDH1 and IDH2. The only sensors proposed to date are the 2D disposable stochastic sensors, which are capable of determining IDH1 and IDH2 in whole blood and tissue samples [11]. There are numerous commercial ELISA kits used for the assay of IDH1 and IDH2 in clinical laboratories as standard methods. The US Food and Drug and Administration (FDA) office recently approved a method for the assay of IDH1 and IDH2 based on PCR analysis [12]. These methods are very expensive and time-consuming; furthermore, extensive processing of the biological sample is needed.

To address the necessities of clinical practice for the molecular recognition and determination of IDH1 and IDH2 in biological samples, we developed a reliable, fast, and cost-effective screening method based on the utilization of stochastic microsensors—the only type of sensors able to perform qualitative and quantitative analysis [13,14,15]. The stochastic microsensors were based on immobilization of a solution of PIX in SWCNT and MWCNT decorated with Cu and Au nanoparticles. Carbon nanotubes (CNT) have the good conductivity (improved by the addition of Cu and Au nanoparticles) and good chemical stability needed to maintain in shape the channels of the stochastic microsensors [16,17]. Protoporphyrin IX is well known for its ability to form the molecular aggregates and stable channels needed for stochastic sensing [18]. 

The main advantage of the proposed 3D stochastic sensors versus the 2D stochastic sensors is that they can be used for more than one month continuously for more than 100 measurements, maintaining a highly reliable profile for the analysis.

## 2. Materials and Methods

### 2.1. Materials and Reagents

Isocitrate dehydrogenase 1, isocitrate dehydrogenase 2, protoporphyrin IX ≥ 95%, copper powder (spheroidal, 10–25 µm, 98%), gold nanoparticles (10 nm diameter, OD 1, stabilized suspension in 0.1 mM phosphate buffer solution (PBS), reactant free), single wall and multiwall carbon nanotube, sodium phosphate monobasic monohydrate, sodium phosphate dibasic heptahydrate, and sodium chloride were procured from Sigma Aldrich (Burlington, Massachusetts, USA). The paraffin oil was purchased from Fluka (Buchs, Switzerland). The IDH1 and IDH2 solutions of different concentrations, 1.00 × 10^−11^ to 10 µg mL^−1^, and 5.00 × 10^−11^ to 50 µg mL^−1^, respectively, were prepared in a PBS 0.15 mol L^−1^ of sodium chloride, using the serial dilution method. For the assay of IDH1, an ELISA kit from Biomatik (Wilmington, DE, USA) was used, while for the assay of IDH2, an ELISA kit from Abbexa (Cambridge, UK) was used.

### 2.2. Instruments and Methods

All experimental measurements were performed at room temperature inside the Faraday cage with an AUTOLAB/PGSTAT 12 (Metrohm) linked to a computer with GPES software version 4.9 (Utrecht, The Netherlands), to record and interpret the measurements. A chronoamperometric method was used for the measurements of t_off_ and t_on_, at a fixed potential (125 mV vs. silver/silver chloride (Ag/AgCl)), with 360 s for the calibration measurements for the analytes as well as for the samples. The electrochemical cell comprised three electrodes: the auxiliary electrode—a platinum wire, the reference electrode—Ag/AgCl, and the working electrode—the stochastic microsensor designed for the molecular recognition of IDH1 and IDH2. 

The 3D microtubes with internal diameters of 25 μm were printed in the laboratory using a Stratasys Objet 24 printer (Rehovot, Israel). The determination of the pH for the buffer solutions was done using a Mettler Toledo pH meter (Columbuo, OH, USA). Deionized water from a Direct-Q3 UV water purification system (Millipore Corporation, Darmstadt, Germany) was used for the preparation of all solutions.

The structural analysis of the active surfaces of the sensors was performed by X-ray diffraction using a PANalytical diffractometer (FEI Company, The Netherlands), with Cu-Kα radiation (λ = 0.15406 nm), and 2*θ* ranging from 20° to 80°. The surface morphology and elemental analysis were performed with a scanning electron microscope (FEI Company, The Netherlands), equipped with an energy-dispersive X-ray detector (EDX). In this case, the working parameters were the following: high voltage (HV), magnification, the working distance (WD), and the used detector (LFD—for low vacuum), with scanning rate not being a given parameter.

### 2.3. Design of 3D Stochastic Microsensors

A total of 100 mg SWCNT powder and MWCNT, respectively, were mixed with 10 μL of Au nanoparticle dispersion which contained 1 mg powder copper, and paraffin oil until two homogeneous pastes were obtained. To obtain the modified pastes, 100 μL solution of PIX (1.00 × 10^−3^ mol L^−1^, prepared in tetrahydrofuran) was added to each of the pastes.

**Figure 1:**
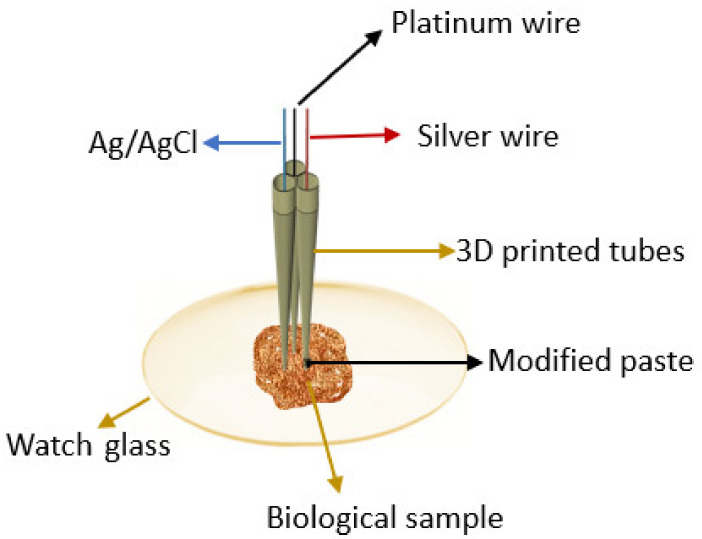
Experimental set-up.

The modified pastes were placed in the 3D microtubes with internal diameters of 25 μm (Figure 1). When not in use, the stochastic microsensors were placed at 4 °C, in a dark place.

### 2.4. Stochastic Mode

**Figure 2:**
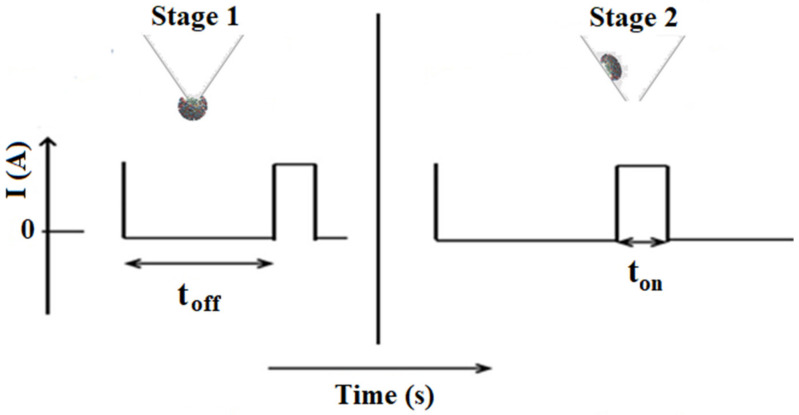
The model for current development in stochastic sensing.

The chronoamperometric method was used for the qualitative and quantitative analysis of IDH1 and IDH2, based on their signatures (t_off_ values), as well as the corresponding t_on_ (which was read in between two t_off_ values) (Figure 2). A constant potential of 125 mV vs. Ag/AgCl at 25 °C was applied for the determination of IDH1 and IDH2. The designed microsensors were introduced into a cell containing analyte solutions of different concentrations. The calibration equations 1/t_on_ = a + b × C_IDH1orIDH2_ were determined using the linear regression method. The concentrations of IDH1 and IDH2 in the biological samples were determined by inserting the values of 1/t_on_ obtained after measuring the biological samples, in the calibration equations.

The principle behind the functioning of the stochastic sensor is based on pore conductivity [19,20,21,22]. The current development of stochastic sensors is shown in Figure 2. Accordingly, with the principles of stochastic methods, all molecules from a solution (sample) may enter into the pores/channels as a function of their sizes, geometry, stereochemistry, and capacity for unfolding. The molecule responsible for the stochastic sensing was the PIX, which in contact with water forms at the membrane–solution interface molecular aggregates presenting the necessary pores for stochastic sensing. After the application of 125mV (Figure 2), the molecular recognition of the biomarkers takes place in two stages. During the first stage, the analyte enters the pore and blocks it, and the current intensity drops to 0 A until the whole analyte enters the pore—the time needed to enter the pore is the signature of the analyte (t_off_ value) and is the qualitative parameter. In the second stage, the interaction of the analyte with the wall of the pore and the redox processes take place during the t_on_—its value is measured in between two t_off_ values and is the quantitative parameter.

### 2.5. Sample Preparation

The proposed 3D stochastic microsensors were used for the molecular recognition and quantitative determination of IDH1 and IDH2 in brain tumor tissue and whole blood samples. The biological samples were collected from confirmed patients with a brain tumor, in accordance with the procedures specified in the Ethics Committee approval number 65573/14.12.2018 awarded by the University Emergency Hospital from Bucharest; written consent was obtained from all patients. All tissues were frozen instantly after resection and stored at temperatures of −80 °C. The whole blood samples were used for the assay of IDH1 and IDH2 immediately after taking them from the patients, without any pre-treatment.

## 3. Results and Discussion

### 3.1. Morphological Characterization of the CNT Pastes

**Figure 3:**
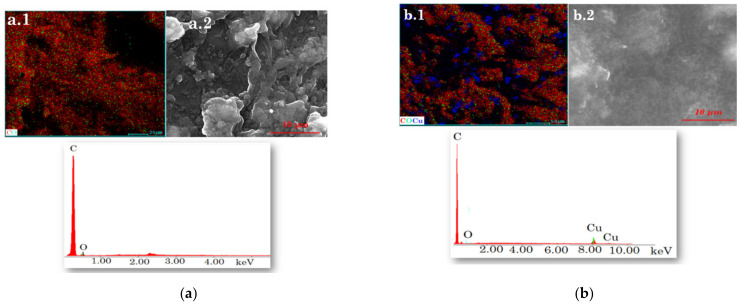
Elemental mapping (**a.1**,**b.1**), surface morphology (**a.2**,**b.2**), and EDX spectrum of the pastes based on: (**a**) CuAuNP-PIX/SWCNT and (**b**) CuAuNP-PIX/MWCNT.

The morphology of the pastes (CuAuNP-PIX/SWCNT and CuAuNP-PIX/MWCNT) that contain the necessary channels for the stochastic response is shown in Figure 3(a.2,b.2). To evaluate the elemental composition, the quantification of the elements, and their distribution in the material, semi-quantitative analysis was performed by EDX. Moreover, from the mapping, the uniform distribution of the elements in both modified pastes may be seen in Figure 3(a.1,b.1).

### 3.2. Response Characteristics of the Stochastic Microsensors

The response characteristics of the stochastic microsensors used for molecular recognition of IDH1 and IDH2 are shown in Table 1. The signatures obtained for IDH1 and IDH2 were different for each of these microsensors, thus demonstrating the ability of the microsensors to perform the molecular recognition of IDH1 and IDH2 in the biological samples.

Utilization of SWCNT or MWCNT did not influence the linear concentration ranges for the assay of IDH1 (1 × 10^−5^–1 × 10^2^ ng mL^−^^1^) and IDH2 (5 × 10^−8^–5 × 10^2^ ng mL^−1^), as well as the limits of quantification for IDH1 (10 fg mL^−1^) and IDH2 (5 × 10^−3^ fg mL^−1^), but it influenced the sensitivity of the proposed stochastic microsensors: the highest sensitivity was obtained when MWCNT was used for the molecular recognition of IDH1 (9.58 × 10^5^ s µg mL^−1^) and IDH2 (1.50 × 10^7^ s µg mL^−1^). Accordingly, the stochastic microsensor of choice for the molecular recognition and quantification of IDH1 and IDH2 is the one based on CuAuNP-PIX/MWCNT.

Compared with the disposable stochastic sensors proposed before [11] (Table 2), a wider linear concentration range and a lower limit of quantification versus the disposable Chitosan/Cu nanolayer-based stochastic sensor was recorded for the assay of IDH1. Moreover, a lower limit of quantification was achieved for the assay of IDH2 with the stochastic sensors based on CNT. Analyses with sensors based on CNT are more cost-effective than those performed using the disposable stochastic sensors because the former can be kept and used continuously for more than one month.

Ten of each type of microsensor were designed and used for 1 month for the assay of IDH1 and IDH2. In this period of time, the sensitivities for IDH1 and IDH2 were recorded. For each type of microsensor, the measurements performed during one day showed that the RSD% values for the variation of the sensitivities recorded for 10 microsensors were 0.10% for IDH1 and 0.15% for IDH2 despite the type of microsensor, proving a highly reliable (reproducible) design of the proposed stochastic microsensors. When used for 1 month, the sensitivity variations were 0.37% for the assay of IDH1 and 0.40% for the assay of IDH2 despite the type of microsensor, proving the stability of the microsensors in time.

The selectivity of the stochastic microsensors is given by the signatures (t_off_ values) recorded for different analytes. The signature of the analyte and the possible interference depends on several factors such as molecule size and conformation, deployment capacity, or speed of going in the channel; thus, the signature can act as an element of molecular recognition, contributing to the qualitative analysis of mixtures. The different signatures obtained for analytes such as IDH1, IDH2, heregulin-α, dopamine, epinephrine, and levodopa proved the selectivity of the proposed stochastic microsensor (Table 3).

### 3.3. Determination of IDH1 and IDH2 in Tumor Brain Tissue and Blood Samples

**Figure 4:**
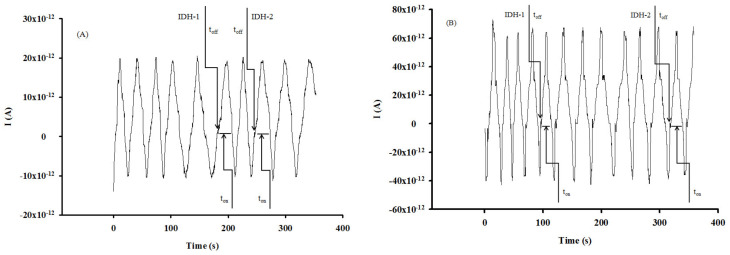
Examples of diagrams recorded for the molecular recognition of IDH1 and IDH2 in brain tumor tissue samples using the stochastic microsensors based on (**A**) CuAuNP-PIX-SWCNT and (**B**) CuAuNP-PIX-MWCNT.

**Figure 5:**
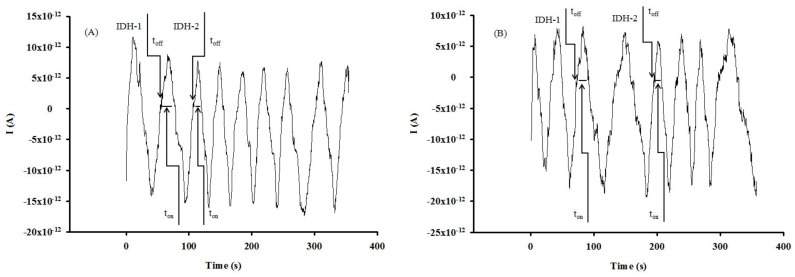
Examples of diagrams recorded for the molecular recognition of IDH1 and IDH2 in whole blood samples using the stochastic microsensors based on (**A**) CuAuNP-PIX-SWCNT and (**B**) CuAuNP-PIX-MWCNT.

Eight brain tumoral tissues and twelve whole blood samples were screened using the proposed stochastic microsensors. Typical diagrams obtained for the screening tests of the brain tumoral tissue and whole blood samples (Figure 4 and Figure 5) were used to perform the molecular recognition of IDH1 and IDH2, based on their signatures, as well as the quantification of IDH1 and IDH2 using the equations of calibration (Table 1). No processing of samples was needed in the case of tissue or whole blood samples; the cell was filled with the sample, and the three electrodes were inserted in the sample. After recording the diagram, the IDH1 and IDH2 were identified accordingly with their signatures (t_off_*)*, and after that, the t_on_ values were read (in between two t_off_ values) and used in the calibration graphs accordingly with the stochastic mode described above, for the quantification of IDH1 and IDH2.

Table 4 and Table 5 show the results obtained for the screening of tumoral brain tissues and whole blood samples. The validation of the proposed stochastic microsensors and the screening method was performed versus the standard method used in clinical laboratories for the determination—ELISA.

A paired *t*-test was performed at a 99.00% confidence level (tabulated theoretical t-value: 4.032) for each type of sample. All calculated t-values (Table 4 and Table 5) were less than 3.00, proving that there is no statistically significant difference between the results obtained using the proposed stochastic sensors. Accordingly, the proposed stochastic microsensors can be reliably used for the molecular recognition and quantification of IDH1 and IDH2 in whole blood and brain tumor tissue samples.

Further, the price of ELISA kits used for IDH1 (more than 800 €/kit) and IDH2 (more than 700 €/kit) is far higher than the price of one stochastic sensor based on SWCNT or MWCNT, which does not exceed 2€ and can be used for more than 100 analyses of tissue and whole blood samples while both IDH1 and IDH2 were determined. 

## 4. Conclusions

The two-3D stochastic microsensors proposed for the molecular recognition of IDH1 and IDH2 were reliably used for screening tests of biological samples such as brain tumor tissue samples and whole blood samples.

The highest sensitivity was recorded when the stochastic microsensor based on MWCNT was used.

Very good correlations between the screening method based on stochastic sensors and ELISA were obtained; this was also proved by the results obtained using the paired *t*-test.

The price of the proposed sensors is 750 times less than the total price of ELISA kits used as a standard method in clinical laboratories.

## Figures and Tables

**Table 1:** The response characteristics of the stochastic microsensors used for the molecular recognition of IDH1 and IDH2.

Stochastic Microsensor Based On	Signature of IDH t_off_ (s)	Linear Concentration Range (ng mL^−1^)	Calibration Equations; The Correlation Coefficient, r *	Sensitivity (s µg mL^−^^1^)	LOQ (fg mL^−1^)
	IDH1				
CuAuNP-PIX/SWCNT	0.7	1 × 10^−5^–1 × 10^2^	1/t_on_ = 0.03 + 1.48 × C; r = 0.9999	1.48	10
	IDH2				
	1.4	5 × 10^−8^–5 × 10^2^	1/t_on_ = 0.03 + 7.30 × 10^4^ × C; r = 0.9999	7.30 × 10^4^	5 × 10^−3^
CuAuNP-PIX/MWCNT	IDH1				
	1.5	1 × 10^−5^–1 × 10^2^	1/t_on_ = 0.04 + 9.58 × 10^5^ × C; r = 0.9989	9.58 × 10^5^	10
	IDH2				
	0.7	5 × 10^−8^–5 × 10^2^	1/t_on_ = 0.16 + 1.50 × 10^7^ × C; r = 0.9999	1.50 × 10^7^	5 × 10^−3^

* <C-concentration> = µg mL^−1^; <t_on_> = s; LOQ—limit of quantification.

**Table 2:** The comparison of stochastic microsensors for the assay of IDH1 and IDH2.

Stochastic Microsensors	Linear Concentration Range (ng mL^−1^)	Sensitivity (s µg mL^−1^)	LOQ (fg mL^−1^)	Reference
Disposable Chitosan/Cu nanolayer		IDH1		[11]
	1 × 10^−4^–1 × 10^2^	1.00 × 10^7^	10^2^	
		IDH2		
	5 × 10^−7^–5 × 10^2^	9.51 × 10^5^	5 × 10^−1^	
Disposable Chitosan/GR * nanolayer		IDH1		
	1 × 10^−8^–1 × 10^2^	3.77 × 10^7^	10^−2^	
		IDH2		
	5 × 10^−8^–5 × 10^2^	1.88 × 10^7^	5 × 10^−2^	
Disposable Chitosan/GR-Cu composite nanolayer		IDH1		
	1 × 10^−5^–1 × 10^2^	2.73 × 10^7^	10^−1^	
		IDH2		
	5 × 10^−8^–5 × 10^2^	4.44 × 10^6^	5 × 10^−2^	
CuAuNP-PIX/SWCNT		IDH1		This work
	1 × 10^−5^–1 × 10^2^	1.48	10	
		IDH2		
	5 × 10^−8^–5 × 10^2^	7.30 × 10^4^	5 × 10^−3^	
CuAuNP-PIX/MWCNT		IDH1		
	1 × 10^−5^–1 × 10^2^	9.58 × 10^5^	10	
		IDH2		
	5 × 10^−8^–5 × 10^2^	1.50 × 10^7^	5 × 10^−3^	

* GR = graphene.

**Table 3:** The selectivity of the stochastic microsensors.

Stochastic Microsensor Based On	t_off_ (s), Signature					
	IDH1	IDH2	Heregulin-α	Dopamine	Epinephrine	Levodopa
CuAuNP-PIX/SWCNT	0.7	1.4	0.2	1.9	3.0	2.5
CuAuNP-PIX/MWCNT	1.5	0.7	1.8	2.4	3.2	2.8

**Table 4:** Determination of IDH1 and IDH2 in brain tumor tissue samples using the stochastic microsensor and ELISA.

Sample No	ng mL^−1^, IDH1			ng mL^−1^, IDH2		
	Stochastic Microsensors Based On		ELISA	Stochastic Microsensors Based On		ELISA
	CuAuNP-PIX-SWCNT	CuAuNP-PIX-MWCNT		CuAuNP-PIX-SWCNT	CuAuNP-PIX-MWCNT	
1	15.26 ± 0.02	16.22 ± 0.03	16.03	26.40 ± 0.02	26.50 ± 0.03	26.85
2	14.03 ± 0.03	14.52 ± 0.02	14.48	42.42 ± 0.03	42.65 ± 0.04	42.82
3	14.76 ± 0.03	16.22 ± 0.04	16.00	27.30 ± 0.03	28.56 ± 0.04	27.85
4	29.97 ± 0.03	29.62 ± 0.02	29.03	35.60 ± 0.04	35.27 ± 0.03	35.57
5	9.19 ± 0.02	9.73 ± 0.03	9.54	63.87 ± 0.05	64.40 ± 0.04	63.90
6	15.26 ± 0.03	15.02 ± 0.04	15.05	34.77 ± 0.03	34.68 ± 0.02	34.70
7	6.90 ± 0.02	6.07 ± 0.03	6.93	22.44 ± 0.04	21.73 ± 0.03	22.48
8	15.85 ± 0.03	15.14 ± 0.02	16.12	23.02 ± 0.02	23.72 ± 0.05	23.80
*t*-test	2.94	1.83		2.87	2.08	

**Table 5:** Determination of IDH1 and IDH2 in whole blood samples using the stochastic microsensor and ELISA.

Sample No	ng mL^−1^, IDH1			ng mL^−1^, IDH2		
	3D Stochastic Microsensors Based On		ELISA	3D Stochastic Microsensors Based On		ELISA
	CuAuNP-PIX-SWCNT	CuAuNP-PIX-MWCNT		CuAuNP-PIX-SWCNT	CuAuNP-PIX-MWCNT	
1	55.25 ± 0.03	53.56 ± 0.02	54.24	98.64 ± 0.02	97.98 ± 0.02	98.70
2	77.25 ± 0.03	73.28 ± 0.05	75.00	70.55 ± 0.02	71.85 ± 0.04	70.88
3	55.24 ± 0.04	53.35 ± 0.05	54.28	97.30 ± 0.03	99.00 ± 0.08	99.02
4	10.84 ± 0.03	9.89 ± 0.03	10.94	34.77 ± 0.05	34.49 ± 0.04	35.00
5	52.75 ± 0.03	53.66 ± 0.05	53.84	56.68 ± 0.03	54.52 ± 0.04	55.94
6	5.36 ± 0.03	5.33 ± 0.02	5.40	21.98 ± 0.02	22.81 ± 0.03	23.03
7	52.97 ± 0.03	53.34 ± 0.04	54.02	44.14 ± 0.02	44.95 ± 0.05	45.00
8	13.04 ± 0.04	13.14 ± 0.02	13.15	20.43 ± 0.03	20.35 ± 0.02	21.00
9	17.50 ± 0.03	17.69 ± 0.04	17.70	33.38 ± 0.04	33.61 ± 0.05	33.54
10	14.49 ± 0.03	13.28 ± 0.05	14.53	35.59 ± 0.03	34.52 ± 0.02	35.80
11	96.48 ± 0.01	96.34 ± 0.02	97.00	102.36 ± 0.03	102.65 ± 0.02	103.00
12	14.11 ± 0.02	14.95 ± 0.03	15.00	26.31 ± 0.02	26.81 ± 0.04	26.90
*t*-test	2.20	1.75		2.56	2.21	

## Data Availability

There are no data available for this paper.
